# Non-conducting functions of ion channels: The case of integrin-ion channel complexes

**DOI:** 10.1080/19336950.2022.2108565

**Published:** 2022-08-08

**Authors:** Elena Forzisi, Federico Sesti

**Affiliations:** Department of Neuroscience and Cell Biology, Robert Wood Johnson Medical School, Rutgers University, NJ, USA

**Keywords:** K^+^ channel, cancer, alzheimer’s disease, traumatic brain injury, actin, apoptosis

## Abstract

Started as an academic curiosity more than two decades ago, the idea that ion channels can regulate cellular processes in ways that do not depend on their conducting properties (non-ionic functions) gained traction and is now a flourishing area of research. Channels can regulate physiological processes including actin cytoskeletal remodeling, cell motility, excitation-contraction coupling, non-associative learning and embryogenesis, just to mention some, through non-ionic functions. When defective, non-ionic functions can give rise to channelopathies involved in cancer, neurodegenerative disease and brain trauma. Ion channels exert their non-ionic functions through a variety of mechanisms that range from physical coupling with other proteins, to possessing enzymatic activity, to assembling with signaling molecules. In this article, we take stock of the field and review recent findings. The concept that emerges, is that one of the most common ways through which channels acquire non-ionic attributes, is by assembling with integrins. These integrin-channel complexes exhibit broad genotypic and phenotypic heterogeneity and reveal a pleiotropic nature, as they appear to be capable of influencing both physiological and pathological processes.

## Non-ionic functions of ion channels

Ion channels comprise a fundamental class of integral membrane proteins spanning all three domains of life. Channels make the lipid membrane permeable to ions and for this reason they are present in virtually any cell type [1]. Historically, channels have been considered in light of their conducting properties that range from shaping electrical impulses in excitable cells, to controlling cell volume, secretion, acidification, and other functions in non-excitable cells. However, during the last two decades, we and others, put forward the concept that channels can affect cellular processes in ways that do not depend on their conducting properties (non-ionic functions) [2–9]. Much progress has been achieved, and it is now established that channels possess non-ionic functions that they exert through a number of different mechanisms.

**Figure 1.**
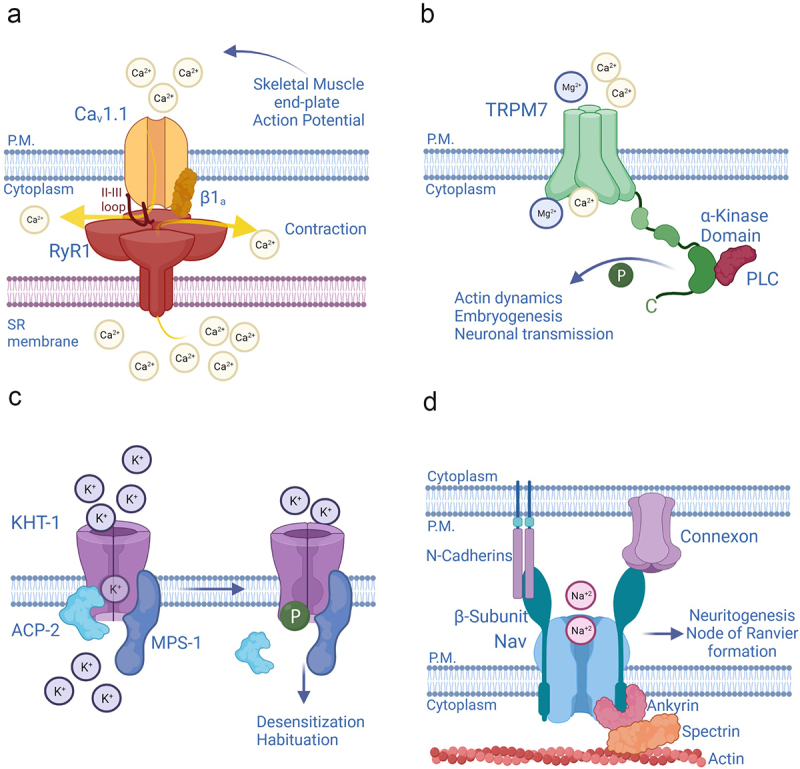
**Ion channels exercise non-ionic functions through multiple mechanisms (a)** Channels perform non-ionic functions through physical coupling with other channels, or posses enzymatic domains in their α-subunits **(b**), or β-subunits **(c)**. In addition to playing canonical regulatory roles, the β-subunits of Nav channels act as adhesion molecules that help forming cell-to-cell contacts, or link the actin cytoskeleton to the extracellular matrix, **(d).**

In skeletal muscle fibers, voltage-gated L-type calcium channels CACNA1S (Cav1.1) operate as voltage sensors of excitation-contraction (EC) coupling. The CACNA1S channels are physically, and functionally paired, to the ryanodine receptors type 1 (RyR1, Figure 1(a)) [10–12]. Thus, CACNA1Ss promote sarcoplasmic calcium increase through two distinct mechanisms: by conducting a calcium current–the effective contribution of which to EC is controversial (see ref. [13]); and by favoring the release of calcium from the stores through their physical, and thus non-ionic, coupling to the RyR1s. Notably, L-type calcium channels and ryanodine receptors are also functionally coupled in the neurons of the brain. The formation of these macromolecular structures is, in turn, helped by delayed rectifier and voltage-gated potassium channel sub-family 2 member 1 (KCNB1, Kv2.1) that acts as scaffolding agent [14–17]. KCNB1 is expressed in several neuron types in the brain and in other organs including eyes, pancreas, gastrointestinal tract, kidney, and female reproductive system, where it presumably carries an important repolarizing potassium current [18]. However, KCNB1 exhibits a broad range of non-ionic functions, such as acting as scaffold protein as noted above that will be discussed throughout this review.

Ion channels have a wide repertoire of mechanisms through which to exert non-ionic functions. For example, the transient receptor potential cation channel melastatin-subfamily, member 7 (TRPM7), a non-selective channel, has enzymatic properties (Figure 1(b)) [3]. The C-terminus of TRPM7 contains a serine/threonine kinase traceable to the α-kinases family [19,20]. TRPM7 can phosphorylate several substrates, including proteins involved in actin dynamics, embryogenesis, and neuronal transmission, underscoring the prominent physiological role of TRPM7 [21–24]. In addition, the α-kinase of TRPM7 indirectly regulates the channel’s ionic properties by modulating its sensitivity to magnesium ions and to magnesium nucleotides [25].

Functional ion channels are seldom the result of a single-gene product. Typically, they are composed of pore-forming or α-subunits, which assemble together to form the pathway for ions (pore) and thus, a conducting channel, and accessory or β-subunits that modulate the properties of the α-subunits. Several accessory subunits of potassium (K^+^) channels, including mammalian Kvβ2, *Drosophila melanogaster* slowpoke channel-binding protein SLOB, and *Caenorhabditis elegans* MiRP K^+^ channel accessory Subunit (MPS-1) have enzymatic attributes [2,4,5]. MPS-1 is an integral membrane protein, homolog to mammalian KCNEs, that has a cytoplasmic domain capable of serine/threonine kinase activity [26,27]. MPS-1 forms complexes with multiple pore-forming subunits including K^+^ channel Voltage-Sensitive Subunit 1 (KVS-1) and K^+^ channel Habituation to Tap subunit 1 (KHT-1) in the nervous system of the worm [9,27]. MPS-1 phosphorylates the α-subunits to decrease their open probability. This mechanism plays an important role in the context of non-associative learning, a universal behavior whereby an organism learns to ignore stimuli that are not important. In the mechanosensory neurons of *C. elegans*, MPS-1 forms a tripartite complex with KHT-1 and with the acid phosphatase ACP-2, which maintains KHT-1 in a basal, de-phosphorylated state (Figure 1(c)) [9,28]. These tripartite complexes mediate the neurons’ response to mechanical stimuli, such as taps to the Petri dish. When the taps are repeated at constant frequency, the animals habituate. The repetitive stimulus triggers the disengagement of ACP-2 from the complex, allowing MPS-1 to phosphorylate KHT-1. This results in a decrease of K^+^ efflux that delays touch-neuron repolarization and, as a consequence, produces temporary desensitization to the mechanical stimuli. Notably, in the neurons of the gigantocellular reticular nucleus, the ACP-2 mammalian homolog, prostatic acid phosphatase (PAP) dephosphorylates KHT-1 homolog, murine KCNC1b, where it may probably underlie adaptation responses [28].

Voltage-gated sodium channels (Nav) acquire non-ionic functions through their β-subunits. These proteins not only modulate the ionic properties of the α-subunits; they also mediate cell-to-cell adhesions by acting as molecular linkers that bridge the actin cytoskeleton to neighboring cells. Thus, the β-subunits connect the Nav with the outside, through interacting with proteins such as neurofascins, N-cadherins, and connexins and with the inside, by attaching to the actin cytoskeleton via assembly with ankyrins (Figure 1(d). The β-subunit promotes direct bonding of Ankyrin G to the Nav α-subunit) [6,29–32]. This non-ionic function likely contributes to the distribution of Nav channels in zones where they accumulate at high densities such as the node of Ranvier and the hillock, and to neuritogenesis, as some β-subunits (β1) augment, whereas others (β2) inhibit neurite’s outgrowth in cerebellar granule neurons [33].

## Integrin-ion channel complexes

A number of ion channels form physical connections with the actin cytoskeleton and with the external environment. However, rather than through their accessory subunits, channels typically achieve those non-ionic functions by interacting with integrins. These are adhesion molecules that connect the extracellular matrix (ECM) to the actin cytoskeleton to regulate the shape, orientation, and movement of cells [34,35]. In addition, integrins engage intracellular signaling pathways, to control cell proliferation (in the absence of integrin-mediated adhesion and growth factors, cells do not commit to enter the cell cycle, [36]), differentiation, survival, and death (anoikis) [37].

Integrin signal transduction is complex and extensively interconnected; therefore, a detailed discussion is beyond the scope of this review (for further readings, we direct the reader to refs. [34,35]). Briefly, in response to anchorage-dependent signals, integrins recruit and/or associate with, the integrin adhesome–a cytoskeletal and signaling complex–to control a multitude of cellular functions [38–40]. Integrins do not possess enzymatic attributes. A fundamental step is the recruitment of Focal Adhesion kinase (FAK), which autophosphorylates Tyr397, creating a binding site for Src kinases. Then, Src, alone or with FAK, phosphorylate several substrates, thereby transducing integrin signals into biochemical events. Integrins promote cell migration by recruiting cytoskeletal linkers, including Talin, and Vinculin, and scaffold/adaptors such as Paxillin and Integrin-linked kinase (ILK) that connect the cytoplasmic tail of integrins to actin filaments. In addition, integrins support cell survival, differentiation, and proliferation, by engaging Ras GTPases, via guanosine-triphosphate exchange factor mSOS. Ras activates several signaling pathways including Mitogen Activated Protein kinase (MAPK) cascades and Phosphoinositide 3 kinase-Protein kinase B-mammalian target of rapamycin (PI3K-Akt-mTOR) signaling that lead to the phosphorylation of cytoplasmic targets and to the enhancement or repression of nuclear transcription.

**Figure 2.**
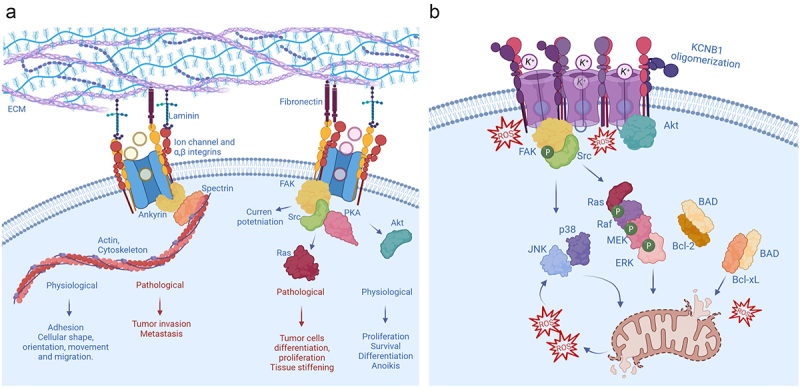
**Non-ionic functions of integrin channel complexes (a)** A widespread mechanism by which channels acquire non-ionic functions is by forming macromolecular complexes with integrins. Generally, these integrin-channel complexes regulate the shape, orientation, and movement of cells through the integrin machinery. In addition, they modulate cell proliferation, differentiation, survival and death, and are therefore implicated in a variety of oncogenic processes of different etiologies. **(b)** Integrin-α5-KCNB1 complexes operate in the neurons of the brain (for simplicity the ECM is not depicted). Under conditions of oxidative stress, the KCNB1 channels form oligomers that trigger apoptosis. The molecular steps underlying this process include the engagement of FAK and Src by the integrins, followed by the activation of a canonical Ras-MAPK cascade. Killer kinases, such as JNK and p38 and caspases execute the apoptotic program. At the same time, these IKCs neutralize a major mechanism of cell survival by sequestering Akt, that cannot be activated (phosphorylated) and released into the cytoplasm, to phosphorylate its multiple substrates including BAD.

Integrin-channel relationships play prominent roles in disease, primarily cancer, given that several aspects of these pathologies, for instance, tumor invasion, differentiation, and metastasis involve cellular functions that require the coordinated action of integrins (Figure 2(a)). In addition, the expression of channels or integrins may adjust the expression of one another to maintain cellular homeostasis. In these cases, increased integrin expression may be associated with channel’s recruitment to the plasma membrane and/or boosted channel activity and *vice versa*. It is also possible that in extreme and/or pathological conditions the increased presence of one may compensate for the absence of the other [41–43]. The first example of cooperation between ion channels and integrins comes from L-type calcium channels expressed in smooth muscle cells of small blood vessels. In rat arterioles, integrin signaling favors vasoconstriction by potentiating CACNA1C (Cav1.2) channels [44–46]. Integrin ligands induce the assembly of integrins α_5_β_1_ with CACNA1C channels to form macromolecular complexes with protein kinase A (PKA), and c-Src tyrosine kinases [47]. Following integrin engagement, c-Src phosphorylates CACNA1C leading to current potentiation that facilitates vasoconstriction [48]. Interestingly, α_5_β_1_ integrins modulate large conductance, calcium-activated K^+^ (BK) channels in the same vessels, through largely similar mechanisms [49,50]. The mechanosensitive ion channel PIEZO1 provides an example of a channel regulating integrin signaling [51]. PIEZO1 is overexpressed in aggressive cancers at focal adhesions where it augments tissue stiffening and tumor cell proliferation [52,53]. An increase in PIEZO1 current causes the activation of integrin-FAK signaling that reinforces tissue hardening. In turn, the firmer mechanical microenvironment boosts PIEZO1 expression and promotes tumor cell proliferation in a sort of auto-catalytic process [54]. Another example of a channel able to modulate integrin function is chloride intracellular channel, CLIC1. This protein promotes integrin-mediated, cell-matrix adhesion and the signaling for cytoskeleton extension during tumor cell migration and invasion [55]. Integrins signal through FAK and the critical role of this protein kinase in promoting cell motility is well established [56,57]. In some cancer types, however, FAK activity is decreased and with that, cellular proliferation and invasion. This inhibition of FAK activity is mediated by the formation of tripartite FAK, integrin-β4, and calcium-activated chloride channel protein (mCLCA1) complexes. Thus, overexpression of the same leads to phosphorylation and inhibition of FAK and ERK proteins [58].

## Integrin-K^+^ channel complexes are widely expressed

Several macromolecular complexes formed by integrins and K^+^ channels, generically named Integrin-K^+^ channel complexes or IKCs, have been identified and characterized [41,43,49,57,59–73]. Arcangeli and colleagues were the first to reveal the existence of pathophysiological links between integrins and K^+^ channels, when they showed that murine erythroleukemia cell adhesion to fibronectin, and neurite outgrowth of neuroblastoma cells were associated with a potassium current, later attributed to the voltage-gated K^+^ channel KCNH2 (synonyms, Kv11.1, HERG, and hERG1) [67,74–78]. Those initial findings were followed by a series of seminal studies that demonstrated that integrin-mediated cell adhesion of KCNH2 promotes cell differentiation. Most importantly, the activation of KCNH2 channels proceeds through integrin-β1 which was demonstrated to physically interact with the channel [70]. Overall, that body of work shows that integrin-β1-KCNH2 complexes integrate the signaling evoked by cell adhesion to the ECM, with the cell differentiation machinery. The pathological implications of integrin-β1-KCNH2 complexes are significant, when one considers that cell differentiation can play a role in oncogenesis. Accordingly, KCNH2 is highly conserved in tumors of different histogenesis, and integrin-β1-KCNH2 complexes play a central role in cancer formation and progression [72,74,76,79,80]. Furthermore, in colorectal cancer cell lines, the binding of fibronectin and collagen I to integrin-β1 at the ECM level promotes the formation of a tripartite complex composed, other than of integrin-β1, of KCNH2 and of the Na^+^/H^+^ antiporter NHE1. The activity of this tripartite complex regulates the cytosolic pH of colorectal cancer cells, thereby contributing to the maintenance of tumor microenvironment [81].

The voltage-gated K^+^ channel KCNB1 forms stable complexes with integrin-α5 in neurons of the brain [62,64]. It is also likely that these complexes exist in other tissues, including the retina and the pancreas, where both KCNB1 and integrin-α5 are present. Studies carried out in heterologous expression systems indicated that the activity of KCNB1 is translated by the integrins into biochemical events–mediated by FAK, Src, Ras GTPases, MAPKs, and protein kinase Akt–to advance the development of actin-rich cellular protrusions in Chinese hamster ovary (CHO) cells, stimulating their motility and to enhance neuritogenesis of neuroblastoma cells (Figure 2(b)) [57,62–65,82,83]. Accordingly, Src-mediated phosphorylation of KCNB1 at Tyr124 is critical for the proliferation and myelination of murine Schwann cells [84,85]. These functions are predominantly non-ionic in nature. Indeed, while certain non-conducting KCNB1 variants implicated in severe epileptic syndromes engage integrin signaling and stimulate cell migration, other (non-conducting) variants that fail to activate the same cascades do not enhance cell migration [63]. Notably, all those variants are associated with large phenotypic heterogeneity [86]. Overall, it appears that integrin-α5-KCNB1 complexes translate membrane excitability into intracellular signals important for cellular plasticity. This implies that when defective, integrin-α5-KCNB1 complexes could cause neurological disease through a variety of mechanisms ranging from impaired conduction to dysregulated integrin signaling. In fact, the non-ionic, pleiotropic, nature of integrin-α5-KCNB1 complexes becomes relevant in neurodegenerative diseases, when neurons are subject to stressful conditions, namely oxidative stress.

## Integrin-α5-KCNB1 complexes trigger programmed cell death

The non-ionic functions of KCNB1 began to unravel after it was discovered that KCNB1 is a pro-apoptotic protein. Aizenman and collaborators showed that cells expressing KCNB1 undergo apoptosis when challenged by oxidants [87]. Subsequent studies revealed that oxidative stress (an imbalance between the oxidants present in the cell and its antioxidant defenses) correlated well with the activation of a number of protein kinases, including p38 MAPK and Src family of tyrosine kinases that phosphorylated KCNB1 at Ser800 and Tyr124 (the latter residue is also responsible for Src-mediated increase of myelination and proliferation of mouse Schwann cells) [84,85,88–91]. The phosphorylation of the channel enhances its interactions with syntaxin accelerating insertion into the plasma membrane [92]. The increased K^+^ efflux that follows, stimulates caspase and nuclease activity and marks a point of no-return toward apoptosis. However, we later discovered that reactive oxygen species (ROS) can directly modify KCNB1 proteins, turning them into aberrant, toxic channels [93]. The journey into what at the time was uncharted territory began when we tested the idea that the excess ROS that build up in aging cells may oxidize K^+^ channels, leading to neuronal failure. For that exploratory inquiry, we took advantage of the simplicity of *C. elegans*, which indeed turned out to be an excellent tool to capture the essence of the problem. Accordingly, the KVS-1 channel, which is a homolog of KCNB1, becomes progressively oxidated at Cys113 in the sensory neurons of aging worms [27,94]. The oxidative modifications alter the gating of KVS-1, which by impairing sensory neuron excitability, leads to behavioral deficit. The cysteine responsible for the functional alterations of the KVS-1 channel is conserved in KCNB1 (Cys73. The KVS-1 channel possesses a 40 amino-acid domain composed of a N-inactivating ball preceded by an N inactivation regulatory domain, called NIRD which modulates the inactivation of the channel [95]). This suggests that also KCNB1 may be susceptible to redox. In fact, oxidants cross-link KCNB1 subunits to each other (oligomers), by inducing the formation of disulfide bridges involving conserved Cys73 [93]. Most importantly, KCNB1 oligomers were detected in the *post mortem* hippocampi of male and female AD donors (83.8 ± 0.79 yrs. average age) where they were significantly more abundant than in age-matched controls (82.5 ± 0.76 yrs.) [64]. Just to give an idea of the extent of KCNB1 oligomerization in the aging human brain, ~40% of KCNB1 channels were found to be oligomerized in control donors, and this number increased to ~75% in Alzheimer’s donors. Similarly, KCNB1 oligomers were found to be ~35% and ~80% in, respectively, 22 month-old control and 3xTg-AD mice [93]. KCNB1 oligomers do not conduct current [93]. Studies in 3xTg-AD mouse model of Alzheimer’s disease–a pathology characterized by extensive oxidative stress–showed that oxidized KCNB1 channels impair neuron repolarization causing hippocampal hyperexcitability [96,97]. However, the toxicity of KCNB1 oligomers does not stem only from their lack of conduction, and is also caused by non-ionic mechanisms. A KCNB1 variant obtained by replacing Cys73 to Ala (C73A) does not form oligomers and conducts normally. Therefore, C73A channels should give rise to an apoptotic current surge in response to an oxidative insult. In contrast, the mutant does not cause cellular death. This implies that the formation of oligomers, rather than KCNB1 current, is the event that triggers the initial pro-apoptotic stimulus.

## Integrin-α5-KCNB1 complexes are implicated in multiple pathologies

Alzheimer’s disease and Traumatic Brain Injury (TBI) provide two well characterized examples of the pleiotropic nature of integrin-α5-KCNB1 complexes. As the brain undergoes degeneration or trauma, these IKCs turn pathogenic by promoting inflammation and apoptosis via integrins and their signaling machinery [64,65,98]. The signaling pathways recruited by integrin-α5-KCNB1 complexes have been characterized in detail. Conformational changes in KCNB1 such as opening and closing leads to the recruitment of FAK. The kinase autophosphorylates Tyr397, creating a binding site for Src family tyrosine kinases. FAK/Src complexes activate small GTPases of the *Ras* sub-family, which in turn, set in motion a canonical MAPK pathway, composed of Rapidly Activated fibrosarcoma (RAF) kinase, Mitogen-Activated Protein Kinase Kinase (MEK) and Extracellular signal-Regulated kinase (ERK) [62,65]. This is followed by the appearance of killer kinases, including C-Jun N-Terminal Kinase (JNK) and presumably kinases implicated in the surge mechanism such as p38 MAPK, caspases and other death proteins, that execute the final steps of the apoptotic program [82,89,98].

## Integrin-α5-KCNB1 complexes keep balance between cell’s life and death

Studies of integrin-α5-KCNB1 complexes expressed in immortal cells demonstrated that the activation of Ras-MAPK signaling represents a causative step toward apoptosis [63,65]. However, also IKCs formed with anti-apoptotic C73A subunits turned out to engage the same Ras-MAPK cascades. The answer to this conundrum is that the difference between IKCs formed with WT and C73A KCNB1 channels resides in the way they regulate active Akt, a major architect of cell survival. Akt keeps apoptosis progression in check through phosphorylating BCL2 associated agonist of cell death (BAD) at Ser136 [99,100]. When BAD is dephosphorylated, it forms heterodimers with Bcl-2 and Bcl-xL, preventing them from inhibiting the release of cytochrome c through the mitochondrial pore [100]. Accordingly, Akt is significantly more active, and with it, BAD is more phosphorylated, in the presence of IKCs formed with C73A mutants compared to WT [63,65]. Furthermore, pharmacological inhibition of Akt abolishes the protective effect of C73A, but when Ras-MAPK signaling is simultaneously inhibited, apoptosis is also suppressed. The activation of Akt occurs at the plasma membrane, where the kinase is sequentially phosphorylated at Thr308 and at Ser473 before being released back into the cytoplasm [101]. An oxidative insult causes selective increase of Akt binding to WT channels that prevents the kinase from being phosphorylated and released into the cytoplasm [65]. At the moment, the causes for the selective affinity of oxidized WT channels for Akt are not known. An important fact to consider, is that KCNB1 oligomers are poorly endocytosed and consequently build up in the plasma membrane [82]. Hence, it is possible that the increased presence of KCNB1 protein at the membrane might enhance the probability of interacting with Akt. Indeed, under normal conditions, a small fraction of KCNB1 channels, either WT and C73A, co-immunoprecipitate with Akt [65]. In summary, the evidence at hand provides a model for the toxicity of integrin-α5-KCNB1 complexes that predicts that these IKCs send apoptotic stimuli via Ras-MAPK cascades, while simultaneously neutralizing the mechanisms of cellular survival.

## Biomedical relevance of integrin-α5-KCNB1 complexes

The elucidation of the non-ionic functions of IKCs carries important biomedical implications, as drugs that impinge on the signaling pathways engaged by these complexes have the potential to ameliorate multiple pathologies. One promising candidate is Dasatinib, a second-generation Src tyrosine kinase inhibitor. Dasatinib is FDA-approved for the treatment of Philadelphia chromosome-positive (Ph+) chronic myeloid leukemia (CML) and acute lymphoblastic leukemia including Central Nervous System CMS (the drug is blood–brain barrier permeable) [102–110]. Dasatinib, reverses cognitive decline in rodent models of AD by decreasing β-amyloid (Aβ) load and neurofibrillary tau tangles (NFT), inflammation, and oxidative stress [64,111–114]. The potential therapeutic effects of Dasatinib in AD stem from its ability to impinge on multiple cellular mechanisms, which share the involvement of Src tyrosine kinases, such as the oxidation of integrin-α5-KCNB1 complexes [64,98]. Combs and colleagues were the first to show that the inhibition of Src tyrosine kinases by Dasatinib acted to reduce brain inflammation and improved cognitive outcome in mouse model of Alzheimer’s disease [112,113]. Work from our lab further indicated that prolonged Dasatinib treatment in 3xTg-AD mice decreased brain inflammation and Aβ load and reduced behavioral deficit caused by the oxidation of integrin-α5-KCNB1 complexes [64]. Dasatinib was also found to significantly decrease inflammation and neurodegeneration caused by oxidation of integrin-α5-KCNB1 complexes in the Lateral Fluid Percussion (LFP) mouse model of brain trauma, a condition that shares with Alzheimer’s disease copious oxidative stress and Aβ plaque formation [115–120]. Orr and colleagues detected a reduction in total NFT density, neuron loss, and ventricular enlargement following Dasatinib+Quercetin (a flavonoid found in many plants and foods) regimen in human Alzheimer’s neurons and in the brains of a mouse model of tauopathy [111]. Others have sought to identify agents targeting the surge mechanism, which relies on the interaction between the C-terminus of KCNB1 and syntaxin. This effort has led to the identification of a small molecule inhibitor (cpd5) of the protein–protein interaction between syntaxin and KCNB1, that has shown some efficacy in ameliorating neuronal loss in middle cerebral artery occlusion mouse model of ischemic stroke [121]. Currently, seven drugs that inhibit *Ras*-MAPK signaling are FDA-approved for the treatment of multiple cancer pathologies, and Akt agonists are being developed [122–125]. It is therefore to be hoped that in the future, some of the drugs that target components of the integrin-α5-KCNB1 complexes signaling machinery could be repurposed for the treatment of diseases, including Alzheimer’s disease, TBI, and stroke.

## Conclusions

In a short period of time, enormous progress has been achieved in our understanding of ion channels and their non-ionic functions. In 20 years, what started as sporadic, anecdotal evidence has become a solid, broad field of research. Channels exert their non-ionic functions through various mechanisms, ranging from physical coupling, to possessing enzymatic features, and many other mechanisms will likely be discovered as our understanding of these proteins progresses. The repertoire of non-ionic functions of ion channels is implicated in a broad range of physiological processes, as fundamental as actin cytoskeleton remodeling and cell migration, differentiation, embryogenesis, excitation-contraction coupling, and learning and memory formation. Consequently, defective non-ionic functions give rise to pathologies, including TBI, Alzheimer’s disease, stroke, and cancer.

Interestingly, channels achieve their non-ionic functions by primarily interacting with integrins. The voltage-gated and delayed rectifier K^+^ channel KCNB1 provides one of the best examples of the broad pathophysiological implications of non-ionic functions of an ion channel. Integrin-α5-KCNB1 complexes are involved in regulating basic cellular processes, and they become toxic in pathological conditions. The elucidation of these mechanisms may provide pharmacological indications that could be quickly translated to human clinical trials.
